# Draft Whole-Genome Sequences of Haemophilus influenzae Biogroup *aegyptius* Strains Isolated from Five Brazilian Purpuric Fever Cases and One Conjunctivitis Case

**DOI:** 10.1128/MRA.00642-19

**Published:** 2019-07-25

**Authors:** Rafaella F. C. Pereira, Luciana S. Mofatto, Ana C. A. Silva, Danilo A. Alves, Daisy Machado, Thaís H. Theizen, Gonçalo A. G. Pereira, Carlos E. Levy, Luciana M. de Hollanda, Marcelo F. Carazzolle, Marcelo Lancellotti

**Affiliations:** a Department of Biochemistry and Tissue Biology, Institute of Biology, State University of Campinas (UNICAMP), Campinas, São Paulo, Brazil; b Department of Genetics, Evolution and Bioagents, Institute of Biology, UNICAMP, Campinas, São Paulo, Brazil; c Faculty of Pharmaceutical Sciences, UNICAMP, Campinas, São Paulo, Brazil; d Department of Clinical Pathology, School of Medical Sciences, UNICAMP, Campinas, São Paulo, Brazil; Broad Institute

## Abstract

Brazilian purpuric fever is a febrile hemorrhagic pediatric disease caused by Haemophilus influenzae biogroup *aegyptius*, a bacterium which was formerly associated with only self-limited purulent conjunctivitis. Here, we present draft genomes of strains from five Brazilian purpuric fever cases and one conjunctivitis case.

## ANNOUNCEMENT

For over a century, Haemophilus influenzae biogroup *aegyptius* was associated only with seasonal epidemics of self-limited purulent conjunctivitis (“pink eye”). In the 1980s, an emergent clone of H. influenzae biogroup *aegyptius* was identified as the etiological agent of Brazilian purpuric fever (BPF), a fulminant pediatric disease characterized by conjunctivitis, high fever, purpura, and sepsis with a fatality rate of 40 to 70% (1, 2). Major outbreaks of the disease occurred from 1984 to 1990 in the state of São Paulo, Brazil. Sporadic cases have been reported in Australia, the United States (2), and, more recently in 2007, in the Brazilian state of Pará (3).

The BPF clone refers to a group of closely similar, but not identical, strains of H. influenzae biogroup *aegyptius* that were associated with the Brazilian cases and have specific properties such as the presence of an approximately 32-kb plasmid referred to as 3031 and a characteristic multilocus enzyme electrophoresis (MLEE) profile (electrophoretic type 2) (2). To date, only one whole-genome sequence of a BPF clone strain is available in GenBank (strain F3031, GenBank accession no. FQ670178), in which were described 21 H. influenzae biogroup *aegyptius*-BPF-specific coding sequences (CDSs) (4). However, the origin and virulence mechanisms of H. influenzae biogroup *aegyptius* associated with BPF still remain a mystery. In this study, we sequenced five additional strains isolated from BPF cases in Brazil and one strain from a conjunctivitis case in the United States.

Strains stored at –80°C were grown on chocolate agar at 37°C with 5% CO_2_. Genomic DNA was extracted using a cetyltrimethylammonium bromide (CTAB) extraction method (5). The DNA libraries were prepared with the Nextera XT DNA library preparation kit (Illumina, CA, USA) and sequenced using the Illumina HiSeq 2000 platform (100-bp paired-end reads). The number of sequenced reads ranged from 30,479,940 to 53,773,000, representing an extremely high sequencing coverage of 1,604× and 2,830×, respectively (see Table 1 for total reads and genome coverage per sample). To reduce coverage, the reads were filtered by quality (Phred quality score of >30), and only a total of 200× coverage for each sample was considered. The reads were *de novo* assembled using Velvet v. 1.2.03 (6), followed by gene annotation using RASTtk (7–9) for exploratory analysis and the NCBI Prokaryotic Genome Annotation Pipeline (PGAP) v. 3.0 (10) for deposition to GenBank. The NCBI PGAP identified a total of 1,957 to 2,056 genes, 1,787 to 1,849 CDSs, 96 to 156 pseudogenes, and 51 to 54 RNA genes. The strain information and genome statistics are listed in Table 1.

**TABLE 1:** Characteristics and genome sequencing and assembly statistics of H. influenzae biogroup *aegyptius* strains

	Data for strain:					
Parameter	F3030	F3039	F3042	F3283	F1946	KC1018
Isolation site	Blood	Blood	Oropharynx	Blood	Skin lesion	Conjunctiva
Genome size (bp)	1,962,639	1,961,979	1,969,657	1,962,133	1,957,726	1,909,282
No. of contigs	67	138	137	130	74	96
*N*_50_	62,606	24,511	24,813	27,365	59,820	39,216
G+C content (%)	38.0	38.0	38.0	38.0	38.0	38.1
No. of genes	2,008	2,045	2,056	2,035	1,987	1,957
No. of CDSs	1,849	1,836	1,847	1,848	1,840	1,787
Total no. of reads	30,479,940	39,906,340	35,636,936	36,692,872	38,647,860	53,773,000
Genome coverage (×)	1,604	2,100	1,875	1,931	2,034	2,830
GenBank accession no.	LNKQ00000000	LNKN00000000	LNKO00000000	LNKP00000000	LNKS00000000	LNKR00000000
SRA accession no.	SRX5957578	SRX5975171	SRX5958526	SRX5957478	SRX5953066	SRX5954865

A nucleotide BLAST search (11) of the assembled contigs showed the presence of the 21 specific H. influenzae biogroup *aegyptius*-BPF CDSs (4) only in the BPF strains. And, as previously described (1), the five BPF strains have the 3031 plasmid (strain F3031, GenBank accession no. AF447808) (12), which is absent in KC1018.

Draft genomes were also submitted to the H. influenzae multilocus sequence typing (MLST) website (https://pubmlst.org/hinfluenzae/) (13). The BPF-associated strains were found to be of sequence type 65 (ST65), while the conjunctivitis strain has the ST72 profile.

The data presented here will be useful for further studies on the genetic characterization of H. influenzae biogroup *aegyptius* associated with BPF.

### Data availability.

The GenBank and SRA accession numbers are given in Table 1.
